# Supplementary material for: Bayesian refinement of protein structures and ensembles against SAXS data using molecular dynamics
Source: PLoS Comput Biol. 2017 Oct 18;13(10):e1005800. doi: 10.1371/journal.pcbi.1005800 (PMC5662244; doi:10.1371/journal.pcbi.1005800)
Supplement: S1 Archive — (BZ2) [file pcbi.1005800.s011.tar.bz2 › gromacs_4.62_bayesian_saxs_refi/share/html/online/files.html]

File formats


| GROMACS File Formats Main Table of Contents |

**VERSION 4.6  
Sat 19 Jan 2013**

---

### Parameter files mdp: run parameters, input for grompp and tpbconv m2p: input for xpm2ps ### Structure files : gro: GROMACS format g96: GROMOS-96 format pdb: brookhaven Protein DataBank format **Generic structure formats:** gro, g96, pdb, tpr, tpb or tpa **Structure+mass(db):** tpr, tpb, tpa, gro, g96 or pdb.: Structure and mass input for analysis tools. When gro or pdb is used approximate masses will be read from the mass database. ### Topology files : top: system topology (ascii) itp: include topology (ascii) rtp: residue topology (ascii) ndx: index file ### Run Input files : tpr: system topology, parameters, coordinates and velocities (binary, portable) tpa: system topology, parameters, coordinates and velocities (ascii) tpb: system topology, parameters, coordinates and velocities (binary) **Generic run input file formats:** tpr, tpb or tpa ### Trajectory files : trj: x, v and f (binary, full precision) trr: x, v and f (binary, full precision, portable) xtc: x only (compressed, portable, any precision) g87: x and v (ascii) gro: x and v (ascii, any precision) g96: x only (ascii, fixed high precision) pdb: x only (ascii, reduced precision) **Full precision formats:** trr or trj **Generic trajectory formats:** xtc, trr, trj, gro, g96, pdb or g87 ### Energy files : ene: energies, temperature, pressure, box size, density and virials (binary) edr: energies, temperature, pressure, box size, density and virials (binary, portable) **Generic energy formats:** edr or ene ### Other files : dat: generic, preferred for input edi: essential dynamics constraints input for mdrun edo: essential dynamics constraints output for mdrun eps: Encapsulated Postscript log: log file map: colormap input for do\_dssp mtx: binary matrix data out: generic, preferred for output tex: LaTeX input xpm: ascii matrix data, use xpm2ps to convert to eps xvg: xvgr input

---

http://www.gromacs.org
